# Atlas-Based Quantification of Cardiac Remodeling Due to Myocardial Infarction

**DOI:** 10.1371/journal.pone.0110243

**Published:** 2014-10-31

**Authors:** Xingyu Zhang, Brett R. Cowan, David A. Bluemke, J. Paul Finn, Carissa G. Fonseca, Alan H. Kadish, Daniel C. Lee, Joao A. C. Lima, Avan Suinesiaputra, Alistair A. Young, Pau Medrano-Gracia

**Affiliations:** 1 Department of Anatomy with Radiology, University of Auckland, Auckland, New Zealand; 2 National Institute of Biomedical Imaging and Bioengineering, Bethesda, Maryland, United States of America; 3 Department of Radiology, University of California Los Angeles, Los Angeles, California, United States of America; 4 Feinberg Cardiovascular Research Institute, Northwestern University Feinberg School of Medicine, Chicago, Illinois, United States of America; 5 The Donald W. Reynolds Cardiovascular Clinical Research Center, The Johns Hopkins University, Baltimore, Maryland, United States of America; University Hospital of Würzburg, Germany

## Abstract

Myocardial infarction leads to changes in the geometry (remodeling) of the left ventricle (LV) of the heart. The degree and type of remodeling provides important diagnostic information for the therapeutic management of ischemic heart disease. In this paper, we present a novel analysis framework for characterizing remodeling after myocardial infarction, using LV shape descriptors derived from atlas-based shape models. Cardiac magnetic resonance images from 300 patients with myocardial infarction and 1991 asymptomatic volunteers were obtained from the Cardiac Atlas Project. Finite element models were customized to the spatio-temporal shape and function of each case using guide-point modeling. Principal component analysis was applied to the shape models to derive modes of shape variation across all cases. A logistic regression analysis was performed to determine the modes of shape variation most associated with myocardial infarction. Goodness of fit results obtained from end-diastolic and end-systolic shapes were compared against the traditional clinical indices of remodeling: end-diastolic volume, end-systolic volume and LV mass. The combination of end-diastolic and end-systolic shape parameter analysis achieved the lowest deviance, Akaike information criterion and Bayesian information criterion, and the highest area under the receiver operating characteristic curve. Therefore, our framework quantitatively characterized remodeling features associated with myocardial infarction, better than current measures. These features enable quantification of the amount of remodeling, the progression of disease over time, and the effect of treatments designed to reverse remodeling effects.

## Introduction

A computational atlas of image-derived shapes refers to an alignment of maps that relate individual anatomical geometry and function to the distribution of biological variations across a population, which can be described at different scales from genotype to phenotype [1]. Atlas-based analyses of patients and healthy volunteers have recently been explored in several different medical areas. For that purpose, large imaging databases, which enable the construction of probabilistic shape atlases for specific organs or diseases, have been established. Atlas-based analysis of brain anatomy and pathology is well advanced (e.g. [2]), including analysis of occipitalization in children [3], and MRI-based probabilistic atlases of neuroanatomy [4]. In the heart, atlas-based analysis has recently shown the potential to reveal new measures of geometry and function [1]. For example, atlas-based methods have been used to quantify subtle differences in heart shape between individuals born prematurely compared with full term age matched controls [5]. In patients with cardiovascular disease, certain changes in heart shape over time, known as remodeling, are indicative of worse prognostic outcome [6]. After myocardial infarction, remodeling associated with an increase in heart size is a predictor of mortality, and remodeling associated with sphericalization of heart shape is linked with decreased survival [7]. However, standard clinical indices used to describe remodeling are typically simple measures of mass and volume, such as end-diastolic (ED) volume (largest volume), end-systolic (ES) volume (smallest volume) or left ventricular mass. These ignore much of the available shape information. We hypothesized that atlas-based analysis of patients with myocardial infarction would enable better quantification of remodeling features associated with myocardial infarction.

The Cardiac Atlas Project (CAP, http://www.cardiacatlas.org) is a world-wide web-accessible resource, comprising a population atlas of asymptomatic and pathological hearts [8]. The CAP facilitates large-scale data sharing of cardiac imaging studies and their corresponding derived analyses that describe the cardiac shape, structure and function across various population groups. The data has been contributed from several studies, including Defibrillators to Reduce Risk by Magnetic Resonance Imaging Evaluation (DETERMINE) [9], comprising patients with myocardial infarction, and the Multi Ethnic Study of Atherosclerosis (MESA) [10], comprising asymptomatic volunteers. We used cases from both studies to examine the principal components of shape variation between the two cohorts, and thereby characterize shape changes associated with myocardial infarction.

Cardiovascular magnetic resonance (CMR) imaging is a non-invasive modality, which provides detailed, quantitative data of the heart structure and function. Compared to other imaging modalities, CMR does not use ionizing radiation, and is not dependent on restricted views of the heart. As a result, many large research studies are using CMR to collect phenotypic data on cardiac disease. Model-based image analyses were developed in the last decades from the availability of large-scale data set of CMR images. This has led to the growing number of statistical analysis applications for cardiac shape and motion [1]. One particular shape representation is a finite element model, which provides an efficient and accurate representation of complex geometries [11]. This method has been shown to provide a compact and powerful representation of shape and function of the LV, and has been validated against ex-vivo LV mass, against manually-drawn contours in patients with regional wall motion abnormalities, and against cardiac output flow in healthy subjects [12], [13]. However, the statistical analysis of shape parameters has previously been limited by the lack of substantial sample size and/or bias between acquisition protocols.

In this study, we applied principal component analysis (PCA) to characterize the cardiac shape features in a large number of CMR cases. PCA is a widely used dimensionality reduction technique, which has been applied to heart shape analysis [14], motion analysis [15], 3D segmentation [16], and population analysis [17] [18]. After extracting shape features using PCA, we applied a logistic regression to analyze the differences between myocardial infarction patients and asymptomatic volunteers. We also compared the performance (goodness of fit) of the model with standard clinical indices, including LV mass and volume. We found that the shape indices derived from the principal components of the shape variation characterized remodeling better than standard LV mass and volume indices.

## Data and Methods

### 2.1 CMR Data

CMR datasets of 300 patients with myocardial infarction from DETERMINE and 1991 asymptomatic volunteers from MESA were obtained from the CAP database for inclusion in this study. These represented a random sample of the MESA baseline and DETERMINE CMR examinations contributed to CAP with local Institutional Review Board approval. This retrospective study was approved by the Health and Disability Ethics Committees of the New Zealand Government, under reference MEC/08/04/052. Informed participant consent compatible with sharing of de-identified data was obtained in writing in all cases. Imaging studies and derived analyses were de-identified, prior to analysis, in a HIPAA compliant manner [US Health Insurance Portability and Accountability Act of 1996 (HIPAA; Pub.L. 104–191, 110 Stat. 1936, enacted August 21, 1996)], annotated using standard ontological schema, stored in a web-accessible picture archiving and communication system database, and analyzed using atlas-based techniques [8]. The asymptomatic cases were regarded as the control group since, at the time of recruitment, they did not present any clinical symptoms of cardiovascular disease [19]. Table 1 shows the cohort characteristics. Patients were taller and heavier than the asymptomatic group, with larger LV mass, end-diastolic volume (EDV), end-systolic volume (ESV), and blood pressures. They were also more likely to be male. The CMR imaging protocol was different between the two cohorts: the DETERMINE protocol used steady state free precession (SSFP) imaging with 10–12 short axis slices and two long axis slices typically with 6 mm thickness, 4 mm gap, field of view 360–400 mm, 256×192 matrix, flip angle 60°, echo time 1.41 ms, repetition time 2.8 ms, with 20–40 frames per slice (temporal resolution <50 ms) and pixel size from 1.4 to 2.5 mm/pixel depending on patient size. The MESA protocol used fast gradient-recalled echo (GRE) imaging with 10–12 short axis slices and one (four chamber) long axis slice with typical parameters 6 mm thickness, 4 mm gap, field of view 360–400 mm, 256×160 matrix, flip angle 20°, echo time 3–5 ms, repetition time 8–10 ms with 20–30 frames per slice (temporal resolution <50 ms) and pixel size from 1.4 to 2.5 mm/pixel depending on patient size.

**Table 1:** Demographics for the MESA and DETERMINE datasets (mean±std).

	Units	DETERMINE	MESA
Sex (Female/Male) ^‡^		60/238	1034/975
Age†	years	62.76±10.80	61.47±10.15
Height^‡^	cm	173.91±9.80	165.97±9.99
Weight†	kg	79.91±28.00	76.75±16.50
Systolic BP	mmHg	127.50±20.14	126.00±22.00
Diastolic BP^‡^	mmHg	73.86±11.34	71.49±10.33
EDV^‡^	ml	196.32±52.94	125.45±31.17
ESV^‡^	ml	118.60±48.86	47.48±18.74
MASS^‡^	g	168.55±41.19	126.24±36.03

† p<0.05; ^‡^p<0.01.

For continuous variables, p values report a Wilcoxon signed-rank test of the null hypothesis. For categorical variables the p-value reports a 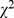 test of the null hypothesis.

### 2.2 Finite Element Modeling

**Figure 1:**
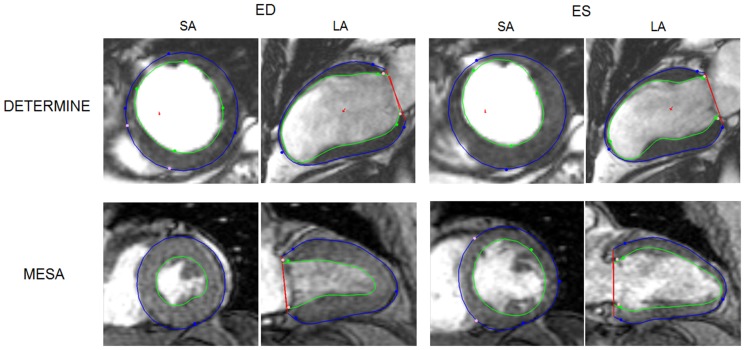
Image and shape differences for volunteers imaged from DETERMINE (top), and MESA (bottom), for the same short-axis (SA), long-axis (LA) planes at end diastole (ED) and end systole (ES). Green and blue contours and markers show the model's endocardial and epicardial boundaries and guide points, respectively. Light color markers denote fiducial landmarks (right ventricular free wall insertion points, mitral valve hinge points) used to define the location of the model shape parameters in consistent positions relative to the anatomy of the heart.

For the MESA cohort, short-axis hand-drawn contours on the inner and outer surfaces of the left ventricle were available from the MESA MRI core laboratory. These contours were fitted by the finite element model by linear least squares as described previously [20]. For the DETERMINE cohort, expert observers performed the analysis using guide-point modeling [13] to interactively customize a time-varying 3D cardiac finite element model of the LV to MR images (Figure 1) using custom software (CIM version 6.0, University of Auckland, New Zealand). LV mass and volume at ED and ES were subsequently calculated from the fitted cardiac LV shape models. The model comprised 16 bicubic finite elements with *C^1^* continuity, (see [12], [13] for details). Briefly, the model was interactively fitted by least-squares optimization to guide points provided by the analyst, as well as computer-generated points calculated from the image using an edge detection algorithm. Automatic feature tracking was used to track points throughout the cardiac cycle using non-rigid registration in both short and long axis images [12]. The model was registered to each case using fiducial landmarks defined at the hinge points of the mitral valve and the insertions of the right ventricular free wall into the inter-ventricular septum. This method has been previously validated against autopsy LV mass, in patients against manually drawn contours and in healthy volunteers against flow-derived measurements of cardiac output [13]. The finite element coordinates were used to provide the atlas coordinates of the LV: each point was assumed to be in approximately the same anatomical location in every heart [21].

### 2.3 Alignment

For statistical analysis, the shape models were evenly sampled at sufficient resolution to capture all the shape features available. The surface sampling process resulted in 2738 Cartesian 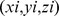 points. A Procrustes alignment method [22] was applied to the sampled point data to determine similarity transformations between shapes: i.e. isotropic scale, translation and rotation. This algorithm finds the optimal scale, rotation matrix and translation vector, which minimizes the overall distance between two sets of points with respect to the Euclidean norm. All LV models from the DETERMINE and MESA datasets were aligned using translation and rotation to their mean shape whenever required. Scale variations were not removed since heart size is a clinical indicator of disease.

### 2.4 Correction of Acquisition Bias

As outlined in Section 2.1, the MESA cohort was acquired using a different imaging protocol (GRE) to the DETERMINE cohort (SSFP). It is known that these two protocols result in small differences in the placement of inner and outer surfaces of the heart. SSFP typically gives rise to larger estimates of left ventricle (LV) cavity volume and smaller estimates of LV mass than GRE. The shape bias has been shown to be regionally variable, and can be effectively removed using a maximum likelihood correction algorithm [23]. Briefly, a transformation between GRE models and SSFP models was learned using data from 40 asymptomatic individuals who were scanned using both protocols. The optimal transformation was found using maximum likelihood methods and was validated previously [23]. All MESA shape models were then transformed using this method, with the transformed shapes then being directly comparable to SSFP models.

### 2.5 Principal component analysis and Logistic regression classification

Principal component analysis [24] is currently one of the most widely used dimension reduction procedures. Using orthogonal transformations, PCA projects the data onto a linear space of maximum-variation directions, known as *modes*. After the projection, the number of modes retained is typically well below the number of original variables, yet still retains a high percentage of the overall variability in the original set. The first mode accounts for as much of the variability in the data as possible, and each succeeding mode in turn has the highest residual variance possible under the linear orthogonality constraint. The coordinates (*x*, *y*, *z*) of the surface sampling points were concatenated into a shape vector. Shape vectors from all cases were formed into a matrix. The eigenvectors of the covariance matrix formed the principal component modes, and their corresponding eigenvalues indicate the proportion of the total variation explained by each mode. Selecting the number of PCA modes to retain in subsequent analysis is contingent on the application. In this paper, enough modes were retained to explain 90% of the total variance. Three PCA cases were considered, the first using only shape vectors at ED, the second using shape vectors at ES, and the third using a combination of ED and ES (ED&ES). The ED&ES PCA was formed by concatenating the shape vectors from ED and ES into a single shape vector.

After PCA, a logistic regression model [25] was used to identify which modes were most associated with the differences between myocardial infarct patients and asymptomatic patients. The weights of the PCA components (up to 90% of the total variability) were used as predictors for classification. In statistics, logistic regression is a type of probabilistic, statistical classification model, which is used to predict a binary response from continuous, binary, or canonical variables. MESA cases (non-patients) were assigned a zero label whereas DETERMINE cases (patients) were assigned a one label. These values were used to obtain the coefficients in the regression models. Thus, the following equation can be used to calculate the probability that a new case belongs to the patient class [26]:
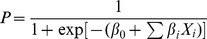
where *P* is the probability of the a certain case belonging to the myocardial infarction set, 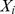 are the values of the predictors, which in our case represent the PCA modes, 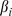 are the coefficient terms of 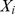, and 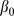 is the intercept. The β terms were found by maximum likelihood estimation. After the coefficients have been estimated, the goodness-of-fit of the resulting model can be examined to determine how well the regression model distinguishes between non-patients and patients. Three common statistics used to quantify the goodness-of-fit of the model are deviance, Akaike information criterion (AIC) and Bayesian information criterion (BIC) [27], [28]:
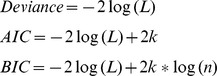
where the *L* represents the log-likelihood of the model (i.e. the value that is maximized by computing the maximum likelihood value for the 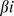 parameters), *k* is the number of estimated parameters and *n* is the sample size. In all three measures, a lower number is indicative of a better model. In addition to these three measures, we also evaluated the area under the curve (AUC) of the receiver operating characteristic curves, since this is also an overall measure of goodness of fit (better models having values closer to 1.0).

## Results

**Figure 2:**
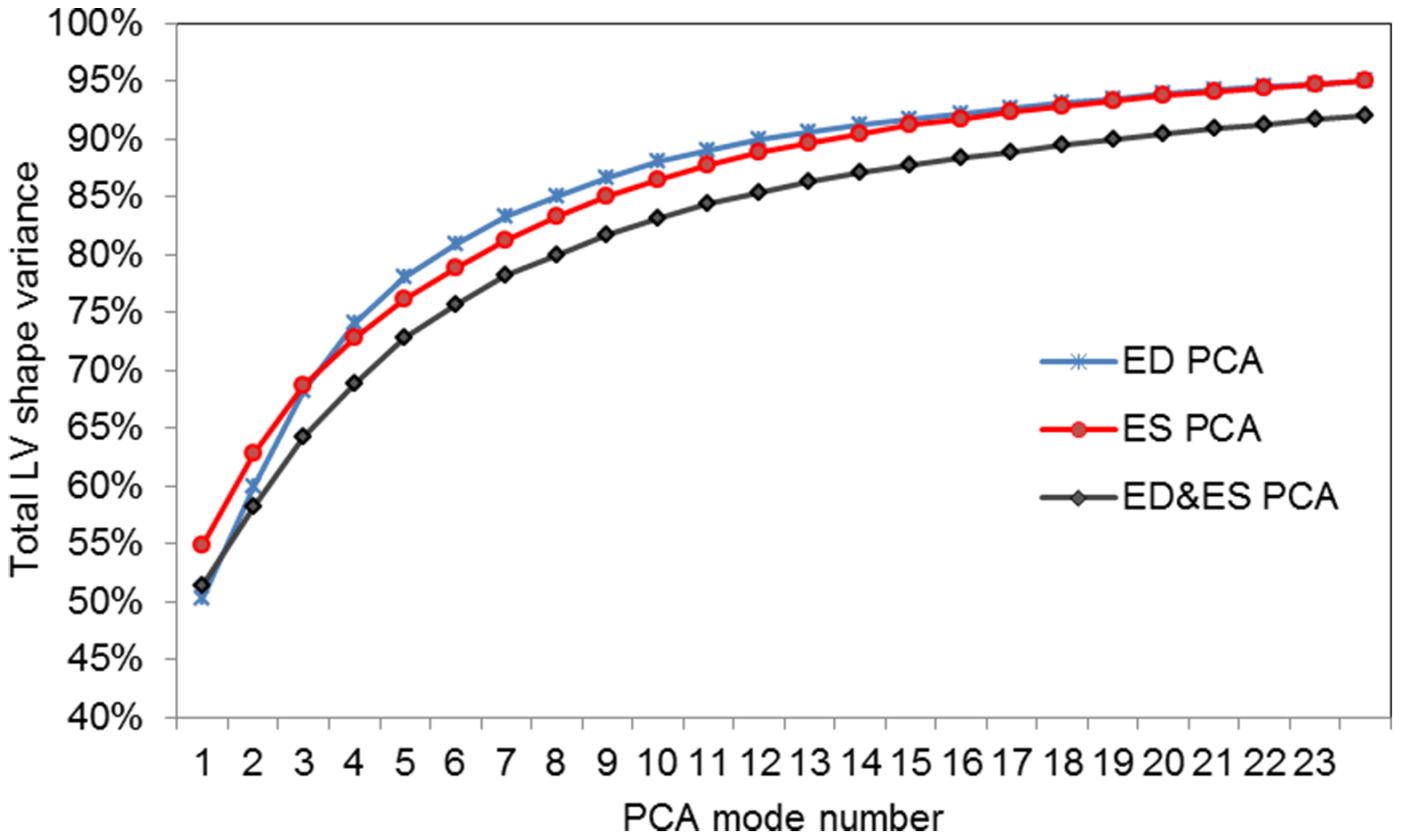
Scree plot of PCA analysis at ED and ES.

**Figure 3:**
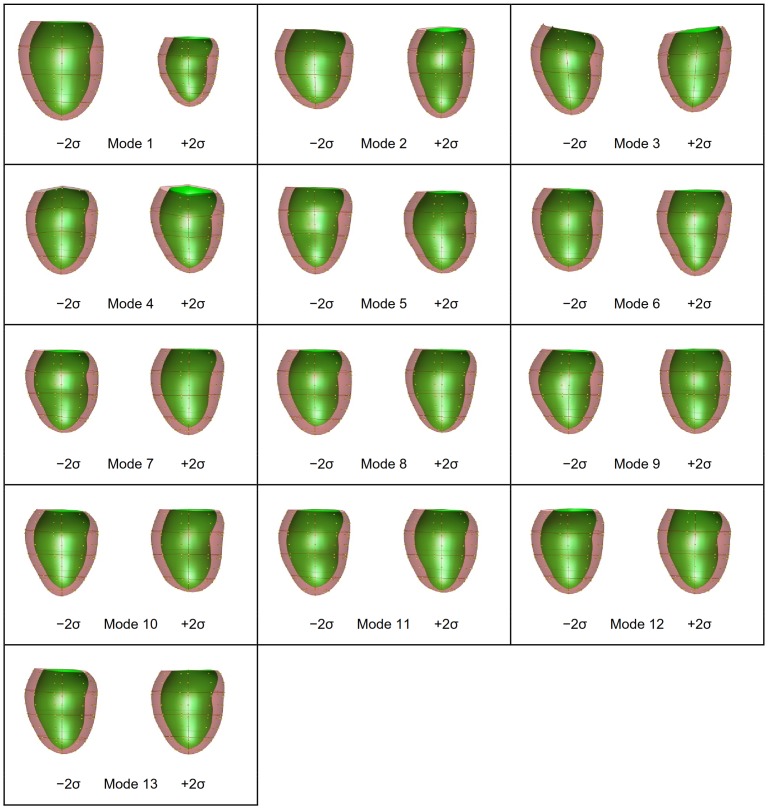
PCA first 13 modes using shape vectors at ED.

**Figure 4:**
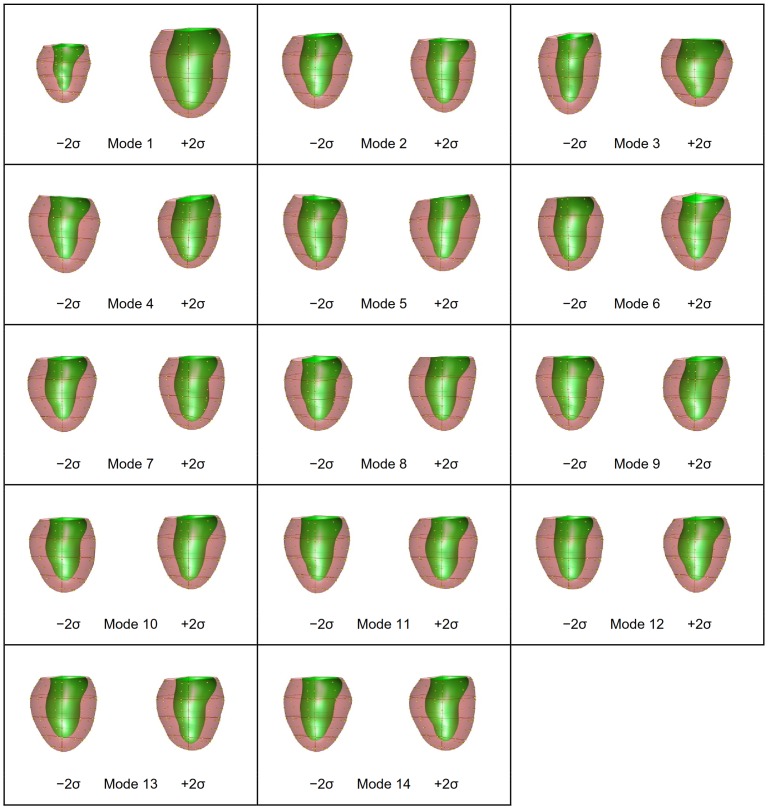
PCA first 14 modes using only shape vectors at ES.

**Figure 5:**
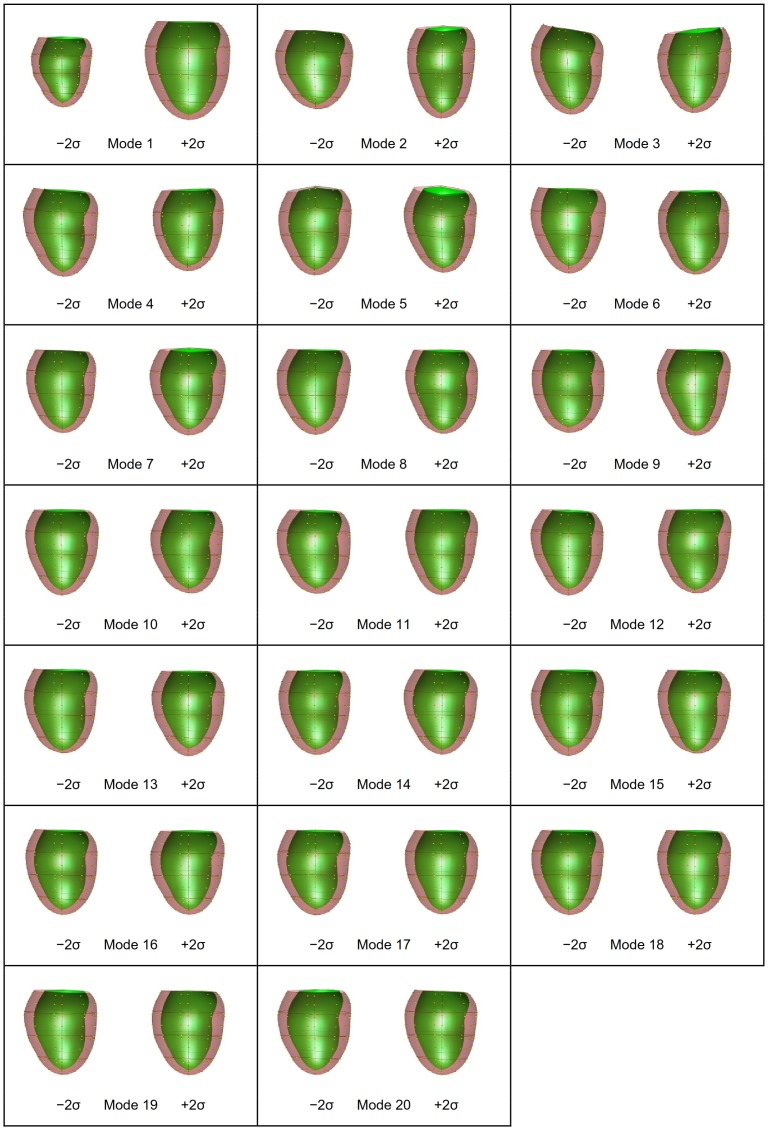
First 20 modes at ED using PCA of a combination of ED and ES.

**Figure 6:**
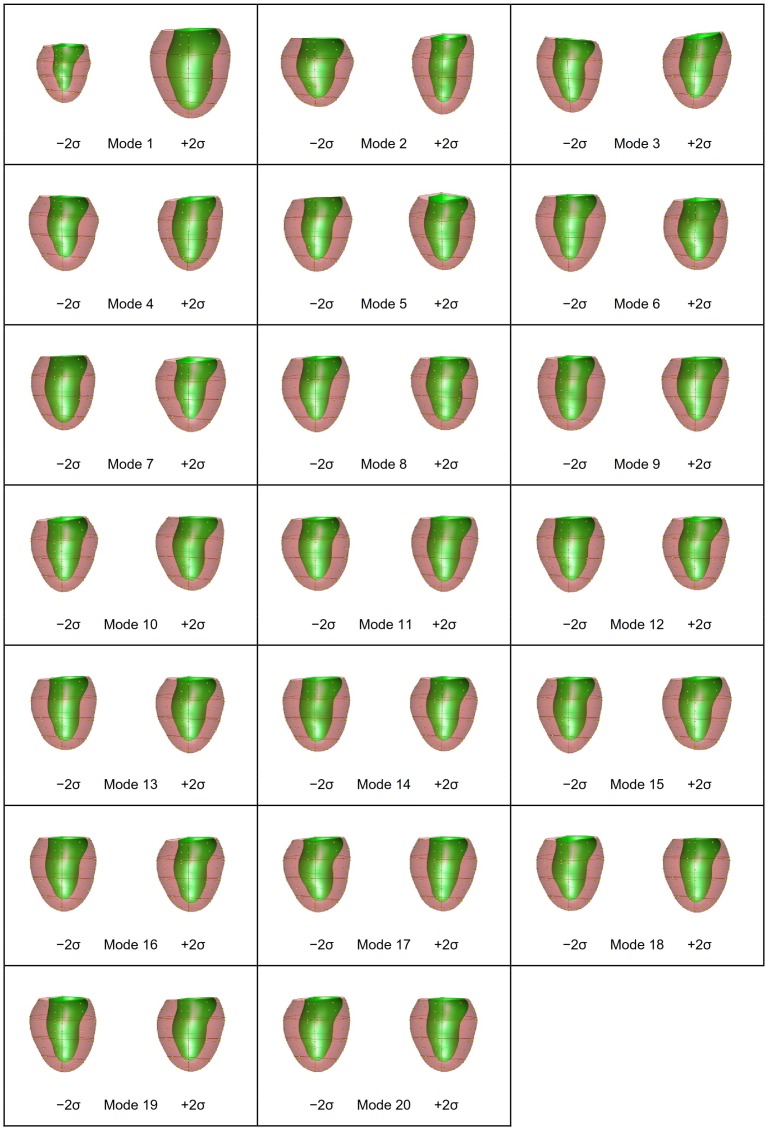
First 20 modes at ES using PCA of a combination of ED and ES.

PCA was performed on the shape models at ED and ES as well as their combination (ED&ES). A scree plot [29] is given in Fig. 2 showing the cumulative variance explained by each mode. The shape variation described by each mode is shown in Fig. 3 for ED, Fig. 4 for ES, and Fig. 5 and Fig. 6 for ED&ES. Although most of the modes do not correspond with traditional shape measures, the first three modes in each case can be understood in terms of commonly used clinical measures of remodeling. Mode 1 explained 50% of the total variance at ED and 55% at ES. In both cases the first mode was primarily associated with the size of the LV. Mode 2 explained 10% of the total variance at ED and was primarily associated with the sphericity of the left ventricle. The third mode of ED was associated with mitral valve orientation. At ES the second mode accounting for 8% of the variance was associated with wall thickening. The third mode at ES was associated with sphericity. We retained 90% of the cumulative variance, which resulted in 13 modes at ED, 14 modes at ES and 20 modes for the ED&ES combination.

Five logistic regression models were studied using all available data. The coefficients (β), standard error, associated p-values, standardized coefficients and odds ratios (OR) were calculated for each model. A p-value of 0.05 or lower was considered significant. The first model included age, sex, height, weight, systolic blood pressure and diastolic blood pressure (Table 1). This was considered the baseline model (Table 2). The second model included the baseline model variables plus 14 ES PCA modes (ES PCA, Table 3). The third model consisted of the baseline variables plus 13 ED PCA modes (ED PCA, Table 4). The fourth model consisted of the baseline variables plus the modes obtained from the ED&ES combination of the ED and ES shape parameters (ED&ES PCA, Table 5). The fifth model included the baseline variables plus the ED volume (EDV), the ES volume (ESV) and the LV mass (MASSVOL, Table 6). The baseline model showed that the age, height and weight were statistically significant predictors of disease (Table 2). These were also significant in the baseline plus ES PCA model (Table 3), along with most of the PCA modes (except modes 5, 8, 11 and 13). The baseline plus ED modes (Table 4) also showed that most PCA modes were significantly associated with disease (except modes 4, 7, 10, 12 and 13). The baseline plus mass and volume model showed that EDV, ESV and MASS were all associated with disease (Table 6).

**Table 2:** Logistic regression analysis of the baseline model.

Parameter	Coefficient	Standard Error	Standardized coefficient	Odds Ratio(OR)	OR 95% Confidence Interval	
Intercept*	−18.8662	1.9036				
Age‡	0.0233	0.0085	0.1308	1.0240	1.0070	1.0410
Sex	0.4107	0.2263	0.1132	1.5080	0.9680	2.3500
Height*	0.0943	0.0111	0.5316	1.0990	1.0750	1.1230
Weight*	−0.0216	0.0046	−0.2148	0.9790	0.9700	0.9880
SBP	0.0045	0.0053	0.0536	1.0040	0.9940	1.0150
DBP	0.0002	0.0105	0.0010	1.0000	0.9800	1.0210

‡ p<0.01 * p<0.0001.

**Table 3:** Logistic regression analysis of the modes at ES.

Parameter	Coefficient	Standard Error	Standardized coefficient	Odds Ratio(OR)	OR 95% Confidence Interval	
Intercept*	−16.8281	4.1446				
Age^‡^	0.0467	0.0178	0.2629	1.0480	1.0120	1.0850
Sex	−0.4471	0.4698	−0.1232	0.6400	0.2550	1.6060
Height†	0.0506	0.0245	0.2851	1.0520	1.0020	1.1040
Weight^‡^	−0.0306	0.0086	−0.3048	0.9700	0.9540	0.9860
SBP^‡^	0.0310	0.0114	0.3732	1.0320	1.0090	1.0550
DBP	−0.0239	0.0206	−0.1378	0.9760	0.9380	1.0170
mode1*	0.0214	0.0018	1.8503	1.0220	1.0180	1.0250
mode2*	0.0209	0.0030	0.7308	1.0210	1.0150	1.0270
mode3*	0.0111	0.0026	0.3281	1.0110	1.0060	1.0160
mode4*	0.0463	0.0049	1.1490	1.0470	1.0370	1.0580
mode5	−0.0011	0.0039	−0.0250	0.9990	0.9910	1.0060
mode6^‡^	−0.0126	0.0044	−0.2509	0.9870	0.9790	0.9960
mode7*	0.0264	0.0043	0.4954	1.0270	1.0180	1.0350
mode8	0.0085	0.0046	0.1508	1.0090	0.9990	1.0180
mode9*	−0.0245	0.0052	−0.3856	0.9760	0.9660	0.9860
mode10*	0.0260	0.0063	0.3877	1.0260	1.0140	1.0390
mode11	0.0054	0.0067	0.0736	1.0050	0.9920	1.0190
mode12^‡^	−0.0242	0.0064	−0.3150	0.9760	0.9640	0.9890
mode13	−0.0022	0.0072	−0.0248	0.9980	0.9840	1.0120
mode14*	0.0386	0.0077	0.4035	1.0390	1.0240	1.0550

† p<0.05; ^‡^p<0.01; * p<0.0001.

**Table 4:** Logistic regression analysis of the modes at ED.

Parameter	Coefficient	Standard Error	Standardized coefficient	Odds Ratio(OR)	OR 95% Confidence Interval	
Intercept	−6.5146	3.5722				
Age*	0.0508	0.0153	0.2859	1.0520	1.0210	1.0840
Sex	−0.4259	0.4037	−0.1174	0.6530	0.2960	1.4410
Height	0.0119	0.0212	0.0674	1.0120	0.9710	1.0550
Weight*	−0.0385	0.0078	−0.3826	0.9620	0.9480	0.9770
SBP	−0.0078	0.0093	−0.0936	0.9920	0.9740	1.0110
DBP	−0.0002	0.0174	−0.0010	1.0000	0.9660	1.0340
mode1*	−0.0212	0.0017	−1.6175	0.9790	0.9760	0.9820
mode2*	−0.0201	0.0024	−0.6924	0.9800	0.9750	0.9850
mode3*	0.0112	0.0025	0.3573	1.0110	1.0060	1.0160
mode4	0.0019	0.0028	0.0497	1.0020	0.9960	1.0070
mode5*	−0.0186	0.0037	−0.4163	0.9820	0.9750	0.9890
mode6*	−0.0557	0.0056	−1.0397	0.9460	0.9360	0.9560
mode7	−0.0061	0.0049	−0.1010	0.9940	0.9840	1.0030
mode8*	0.0528	0.0067	0.7886	1.0540	1.0400	1.0680
mode9^‡^	−0.0142	0.0045	−0.1954	0.9860	0.9770	0.9950
mode10	0.0112	0.0069	0.1442	1.0110	0.9980	1.0250
mode11*	0.0875	0.0102	0.9628	1.0910	1.0700	1.1140
mode12	0.0006	0.0071	0.0062	1.0010	0.9870	1.0150
mode13	0.0105	0.0074	0.0929	1.0110	0.9960	1.0250

† p<0.05; ^‡^p<0.01; * p<0.0001.

**Table 5:** Logistic regression analysis of the modes combined ED and ES.

Parameter	Coefficient	Standard Error	Standardized coefficient	Odds Ratio(OR)	OR 95% Confidence Interval	
Intercept	−15.9741	4.7246				
Age	0.0384	0.0214	0.2157	1.0390	0.9960	1.0840
Sex	−0.2512	0.5221	−0.0692	0.7780	0.2800	2.1640
Height	0.0530	0.0285	0.2991	1.0540	0.9970	1.1150
Weight*	−0.0371	0.0097	−0.3694	0.9640	0.9450	0.9820
SBP	0.0195	0.0137	0.2345	1.0200	0.9930	1.0470
DBP	−0.0145	0.0246	−0.0834	0.9860	0.9390	1.0340
mode1*	0.0160	0.0015	1.8174	1.0160	1.0130	1.0190
mode2*	−0.0122	0.0021	−0.5272	0.9880	0.9840	0.9920
mode3	−0.0024	0.0025	−0.0971	0.9980	0.9930	1.0020
mode4*	0.0438	0.0046	1.5528	1.0450	1.0350	1.0540
mode5†	0.0068	0.0029	0.2227	1.0070	1.0010	1.0130
mode6	0.0012	0.0037	0.0329	1.0010	0.9940	1.0080
mode7†	−0.0314	0.0045	−0.8131	0.9690	0.9610	0.9780
mode8†	0.0089	0.0043	0.1963	1.0090	1.0000	1.0180
mode9	−0.0023	0.0045	−0.0479	0.9980	0.9890	1.0060
mode10	0.0096	0.0050	0.1906	1.0100	1.0000	1.0200
mode11	−0.0006	0.0051	−0.0115	0.9990	0.9890	1.0090
mode12^‡^	−0.0217	0.0056	−0.3548	0.9790	0.9680	0.9890
mode13*	−0.0263	0.0067	−0.4121	0.9740	0.9610	0.9870
mode14*	0.0264	0.0065	0.3784	1.0270	1.0140	1.0400
mode15*	0.0293	0.0086	0.4079	1.0300	1.0130	1.0470
mode16^‡^	0.0195	0.0071	0.2479	1.0200	1.0060	1.0340
mode17	0.0092	0.0070	0.1122	1.0090	0.9960	1.0230
mode18	0.0076	0.0073	0.0935	1.0080	0.9930	1.0220
mode19	−0.0126	0.0082	−0.1451	0.9870	0.9720	1.0030
mode20	0.0033	0.0080	0.0371	1.0030	0.9880	1.0190

† p<0.05; ^‡^p<0.01; * p<0.0001.

**Table 6:** Logistic regression analysis of the modes for LV volume and Mass.

Parameter	Coefficient	Standard Error	p value	Standardized coefficient	Odds Ratio(OR)	OR 95% Confidence Interval	
Intercept	−14.6045	2.9025	<.0001				
Age	0.0246	0.0128	0.0538	0.1384	1.0250	1.0000	1.0510
Sex	−0.2043	0.3522	0.5619	−0.0563	0.8150	0.4090	1.6260
Height^‡^	0.0650	0.0176	0.0002	0.3664	1.0670	1.0310	1.1040
Weight*	−0.0321	0.0068	<.0001	−0.3197	0.9680	0.9560	0.9810
SBP	0.0126	0.0080	0.1136	0.1515	1.0130	0.9970	1.0290
DBP	−0.0138	0.0158	0.3826	−0.0792	0.9860	0.9560	1.0170
EDV^‡^	−0.0245	0.0070	0.0005	−0.5374	0.9760	0.9630	0.9890
ESV*	0.1175	0.0099	<.0001	2.0280	1.1250	1.1030	1.1470
MASS^‡^	−0.0185	0.0048	0.0001	−0.3894	0.9820	0.9730	0.9910

† p<0.05; ^‡^p<0.01; * p<0.0001.

The standardized coefficients show which modes have greater effect on the probability that the case is a patient. Mode 1 and mode 4 have greater effect in the classification model at ES. Mode 1, mode 6 and mode 2 have greater effect in the classification model at ED. EDV, ESV and MASS are highly related with the disease, according to Table 6. The Odds ratios were relative measures of the effects of the shape indicators between the myocardial infarction patients and the normal people. Some shape indicators (OR>1), for instance, the mode 1 and mode 4 in the ES model (Table 3) and ED&ES model (Table 5) show higher odds of myocardial infarction than others. Some shape indicators (OR<1) show lower odds of the disease, for example, mode 1 and mode 6 in the ED model (Table 4). There are several modes whose OR is not significant as their confidence intervals overlap the null value (OR = 1).

**Figure 7:**
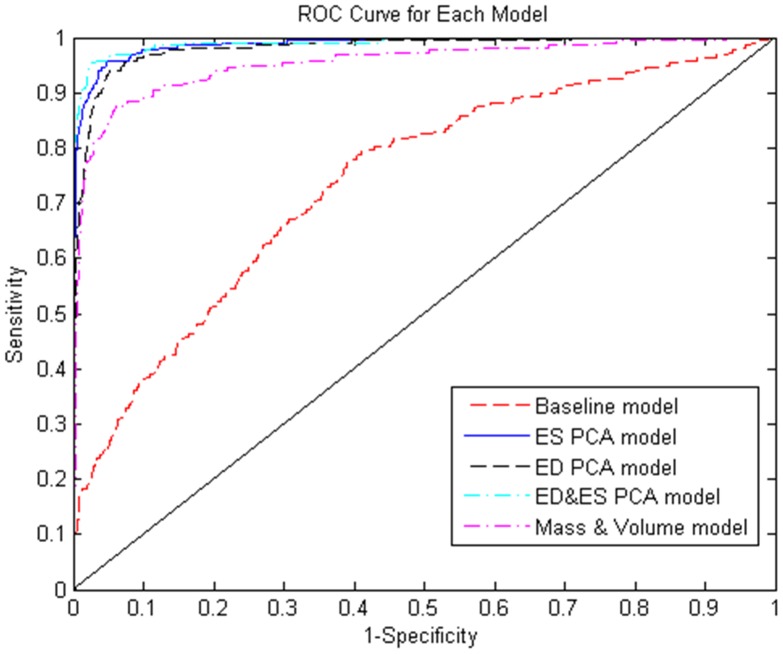
ROC curve for the logistic regression classification for each model.

The goodness of fit was compared between the five models with the indices of Deviance, AIC, BIC and AUC of each model, which are listed in Table 7. Overall, all the PCA mode models as well as the mass-volume model showed good performance. The ED&ES PCA model achieved the best performance in terms of Deviance, AIC, SC, and AUC values, followed closely by the ES PCA model and the ED PCA model. All PCA models better characterized patients from non-patients than traditional mass and volume measures. The ROC curves are shown in Fig. 7.

**Table 7:** Comparison of the five logistic models.

	Deviance	AIC	BIC	AUC
Baseline Model	1254.44	1268.44	1308.34	0.7404
MASSVOL Model	602.641	622.641	679.644	0.9530
ED PCA Model	411.088	451.088	565.094	0.9810
ES PCA Model	319.881	361.881	481.587	0.9883
ED&ES PCA Model	260.753	314.753	468.661	0.9905

## Discussion

We have proposed an atlas-based disease analysis framework by means of shape parameters from LV finite element models with a large number of subjects. The framework consisted of three steps: (1) fitting a finite element model to the LV MR images, (2) principal component analysis of the aligned shape parameters, and (3) quantification of the association with disease using logistic regression. We hypothesized that patients with myocardial infarction have significant shape differences with respect to the normal population, due to cardiac remodeling. The results supported this hypothesis, with most modes significantly associated with disease. The PCA analysis also performed better than traditional indices of remodeling (mass and volume). This method can therefore be used as a clinical tool for the characterization of the patterns of change associated with remodeling. These methods can also be used to track individual patients over time, by quantifying the degree to which their shape modes conform to the remodeling spectrum. Patients who are moving toward the adverse side of the spectrum may benefit from more aggressive treatment regimes. Conversely, the reverse remodeling associated with treatment can also be quantified. Although in this study we applied the method to patients with myocardial infarction, this framework is generalizable to any disease group.

Note that we did not attempt to correct for colinearity between EDV, ESV and Mass in the MASSVOL model, or between SBP and DPB in the baseline model. EDV and ESV were strongly correlated (Pearson coefficient ρ = 0.911, p<0.05), as were ESV and Mass (Pearson coefficient ρ = 0.664, p<0.05), which would affect these coefficient estimates and odds ratios in the model. However, all three were input together in the MASSVOL baseline model to show the combined goodness-of-fit of traditional indicators, in order to assess the improvement given by the PCA modes. SBP, DBP (Pearson coefficient ρ = 0.604, p<0.05) and other baseline variables were included in all the regression models to control for any differences between the patient and asymptomatic groups (Table 1).

The finite-element method is a powerful representation of the LV model, which also provides traditional indicators such as volume and mass. This method has been used to characterize cardiac motion [30], [31] and deformation in a variety of disease groups [32]. Extensions to the right ventricle and atria have also been proposed [33], [34]. In this study, we have limited the application of these models to the description of shape; however, these models also have the capability of simulating the excitation, contraction and relaxation of cardiac mechanics [35].

PCA clusters the variability of the finite element models into orthogonal modes that can be interpreted from a global shape point of view. In [5], PCA was used to determine the shape differences between people born pre-term and people born full-term. PCA has the disadvantage that the modes are in general difficult to interpret from a clinical perspective. However, in the present study the first three modes were associated with well understood clinical indicators such as size or sphericity. Interestingly, both size and sphericity are associated with adverse outcomes after myocardial infarction [7], [36], [37]. In the current study, the ED_ES PCA regression model performed the best with an AUC of 0.9905. Adding ESV, EDV and MASS into this model did not improve this performance greatly (results not shown), indicating that the discriminatory information included in these mass and volume measures are already captured in the ED&ES PCA model. Adding stroke volume or ejection fraction to the MASSVOL model also did not improve the results greatly, since these are very dependent on the ESV and EDV already in the model.

Although heart size is known to be dependent on patient body habitus, we did not correct the shape vectors for height or weight, as done clinically using indexing methods. This was because the baseline model already included height and weight, so all PCA logistic regression analyses were automatically corrected for height and weight.

Further work is needed in several areas. Although PCA is one of the most common dimensionality reduction techniques, other techniques may be more appropriate, such as independent component analysis [18]. Secondly, logistic regression classification method is only one of many methods which can be used for the characterization of disease. For example, in [38] a three-dimensional cortical gray matter density map was established and validated using sparse multinomial logistic regression in the classification of schizophrenia. In [39], a maximum a posteriori classifier was used to distinguish brain tissue types. Expectation-maximization (EM) [40] and k-Nearest-Neighbor [41] classification have been successfully applied to evaluate brain tumors from MRI. Neural networks and support vector machines have been used to identify brain structures with MRI [42] and to predict wall motion scores [43]. Evaluation and comparison of these methods for the evaluation of cardiac disease should be performed. Thirdly, the transformation from GRE to SSFP models was learned using 40 normal volunteers. While [23] showed that this was sufficient to robustly characterize the transformation, more cases would provide a greater variation of heart shape and might improve the transformation parameters.
